# Simultaneous Determination of Seven Antibiotics and Five of Their Metabolites in Municipal Wastewater and Evaluation of Their Stability under Laboratory Conditions

**DOI:** 10.3390/ijerph182010640

**Published:** 2021-10-11

**Authors:** Sheng Han, Xinyue Li, Hongmei Huang, Ting Wang, Zhenglu Wang, Xiaofang Fu, Zilei Zhou, Peng Du, Xiqing Li

**Affiliations:** 1 Laboratory of Earth Surface Processes, College of Urban and Environmental Sciences, Peking University, Beijing 100871, China; hans@pku.edu.cn (S.H.); 1701214469@pku.edu.cn (H.H.); wangting7@pku.edu.cn (T.W.); fxiaof@pku.edu.cn (X.F.); 2 Development Research Center, Ministry of Water Resources of China, Beijing 100036, China; lixinyue@waterinfo.com.cn; 3 College of Oceanography, Hohai University, Nanjing 210098, China; 20190039@hhu.edu.cn; 4 Hubei Provincial Academy of Eco-Environmental Sciences, Wuhan 430070, China; 1601214925@pku.edu.cn; 5 Beijing Key Laboratory of Urban Hydrological Cycle and Sponge City Technology, College of Water Sciences, Beijing Normal University, Beijing 100875, China; dup@bnu.edu.cn

**Keywords:** antibiotics, metabolites, simultaneous analysis, stability, wastewater, human health

## Abstract

The selection and spread of antibiotic resistance poses risks to public health by reducing the therapeutic potential of antibiotics against human pathogens. Wastewater-based epidemiology (WBE) is potentially the most reliable approach to estimate antibiotics use. Previous WBE studies used parent antibiotics as biomarkers, which may lead to overestimation since parent antibiotics may be directly disposed of. Using metabolites as biomarkers can avoid this drawback. This study developed a simultaneous solid-phase extraction coupled with ultra-high-performance liquid chromatography tandem mass spectrometry method for analyzing 12 antibiotics and human metabolites in wastewater to help assess health risk. Optimum conditions were achieved using a PEP cartridge at pH 3.0. The extraction efficiencies were 73.3~95.4% in influent and 72.0~102.7% in effluent for most of the target analytes. Method detection limit ranged from 0.1 to 1.5 ng/L for influent wastewater and 0.03 to 0.7 ng/L for effluent wastewater. A stability experiment showed that sulfonamide parents and their metabolites were stable at 4 °C, −20 °C and −80 °C, while macrolides metabolites were more stable than their corresponding parents at 4 °C and −20 °C. Finally, the method was applied to measure these analytes in wastewater samples collected from three Beijing WWTPs and to derive apparent removal rates. All metabolites were detected in wastewater samples with concentrations ranging from 1.2 to 772.2 ng/L in influent, from <MDL to 235.6 ng/L in effluent. The apparent removal rates of five metabolites were above 72.6%. These results set a solid foundation for applying WBE to evaluate antibiotics use and its public health effects.

## 1. Introduction

Antibiotics are one of the important pharmaceuticals since they are widely used for treating infectious diseases of both humans and animals, as well as the feed additives to promote the growth of farming animals [1,2,3]. Clinic evidences have revealed that antibiotics could not be thoroughly metabolized. Most of them were excreted through feces and/or urine as the mixtures of drugs and their metabolites [4], and then discharged to municipal wastewater treatment plants (WWTPs) [5,6,7]. These compounds could not be eliminated completely via the traditional treatment process in WWTPs. The residues of them might contaminate the aquatic environment system [7,8,9], which may pose adverse effects on ecosystems and human health owning to the increase of allergies in humans and the spread of antibiotic resistance [10,11]. Due to these adverse effects, it is important to estimate the consumption and emission levels of antibiotics in a specific region or country. In the literature, a number of studies did attempt to estimate the consumption of antibiotics by different models [12,13,14]. However, these studies all had fundamental limitations due to lack of basic information, sales, and/or census data.

These limitations may be overcome by wastewater-based epidemiology (WBE), a powerful approach for drug consumption estimation. This approach back-calculates drug consumption based on the concentrations of specific biomarkers in wastewater, the flow of wastewater, the population of the service area, and correction factors that take into account stability and excretion rates of the markers [15]. Compared to traditional methods, this approach can provide objective and near real-time information about the consumption of the population. Due to these advantages, this approach has been applied in many countries in Europe [16,17], North America [18], Oceania [19], and Asia [20,21,22] to monitor drug abuse in these regions. Following successful application of WBE for illicit drug consumption estimation, it has been used to estimate consumption of licit drugs, such as lamivudine (antiviral drug) [23], metformin (antihypertensive drug) [24,25], fexofenadine, and cetirizine (antihistamine drug) [26].

Recently, WBE has been also applied to estimate antibiotics use in several cities in China and Sri Lanka, respectively [27,28,29]. However, all of these three studies chose parent compounds of antibiotics as biomarkers to estimate its consumption, which can cause over-estimation in consumption since antibiotics may well be disposed directly into sewage [30]. Thus, in order to improve the accuracy of estimation, it is necessary to develop methods to determine antibiotic metabolites concentrations in wastewater. To date, many studies measured antibiotics concentrations using solid-phase extraction (SPE) coupled with high performance liquid chromatography tandem mass spectrometry (HPLC-MS/MS) [31,32,33], whereas the methods for determining metabolites were rarely reported. To our knowledge, only a few papers developed analytical methods for detecting sulfonamides metabolites in the influent wastewater [34,35,36], and no studies have investigated other categories metabolites. To apply WBE for monitoring consumption of more antibiotics, it is imperative to develop sensitive and fast analytical methods for other antibiotics and its metabolites.

Furthermore, stability of compounds in wastewater is one of the key factors in selecting biomarkers for consumption estimation [37]. The previous two studies mentioned that many antibiotics were stable in wastewater [38,39], but showed no experimental data. Zhang et al. [28] only reported the stability of antibiotic parents at 4 °C for 24 h under laboratory conditions. Therefore, to accurately back-calculate antibiotics consumption using WBE, it is necessary to evaluate the stability of antibiotics and metabolites during the sampling and storage period.

The objective of this study was to develop a reliable and sensitive method for simultaneously determining antibiotics and metabolites using SPE coupled with UPLC-MS/MS. The linearity of calibration curve, recovery and precision, matrix effects, and method detection limits were all assessed to ensure the applicability of the developed method. The stability of antibiotics and metabolites was examined under different preservation conditions in laboratory. The analytical method was applied to determine the concentration levels of selected antibiotics and their human metabolites in wastewater samples from Beijing WWTPs.

## 2. Materials and Methods

### 2.1. Chemicals and Reagents

Representative antibiotics and their metabolites were selected as target analytes according previous references [27,28,29]. Seven antibiotics, sulfapyridine (SPY), sulfamethoxazole (SMX), roxithromycin (RTM), azithromycin (ATM), trimethoprim (TMP), clarithromycin (CTM), lincomycin (LIN), five human metabolites, N^4^-acetyl sulfapyridine (N-SPY), N^4^-acetyl sulfamethoxazole (N-SMX), N-demethyl roxithromycin (N-RTM), descladinose azithromycin (Des-ATM), 4-hydroxy trimethoprim (4-TMP), and their corresponding deuterated analogs (utilized as internal standards, IS) were all purchased from Toronto Research Chemicals (North York, ON, Canada), with details listed in Table S1. The structures of these antibiotics and metabolites were shown in Figure S1. The stock solutions of target analytes and IS were prepared by dissolving each compound in methanol at a concentration of 100 mg/L, and then stored in refrigerator at −20 °C. HPLC grade of acetonitrile and methanol was obtained from Fisher Scientific (Waltham, MA, USA). Sulphuric acid (AR), methanol (AR), were all purchased from Beijing Chemical Works (Beijing, China). HPLC grade of formic acid, ammonium formate, ammonium acetate and ammonium hydroxide were obtained from CNW Technologies GmbH (Düsseldorf, Germany). Ultrapure water was prepared using a Milli-Q ultrapure system (Millipore, MA, USA). 

As for cartridges selection, based cartridges and sorbent materials adopted in previous publications (e.g., polystyrene and divinylbenzene, N-vinylpyrrolidone and divinylbenzene bonding with sulfonate group and hydrophilic N-vinylpyrrolidone and lipophilic divinylbenzene) [32,35,36], in this study, three different cartridges, namely Cleanert PEP cartridges (60 mg, 3 mL) (PEP) (Agela Technologies, Tianjin, China), Oasis HLB cartridges (60 mg, 3 mL) (HLB), and Oasis MCX cartridges (60 mg, 3 mL) (MCX) (Waters Corporation, Milford, MA, USA), were tested before choosing suitable SPE cartridges. The details of selected SPE cartridges are listed in Table S2. 

### 2.2. Sample Collection

Influent and effluent wastewater samples were collected from three WWTPs in Beijing (named as BJ-1, BJ-2 and BJ-3). These WWTPs mainly receive domestic waters at an average flow rate of 80,000, 532,000 and 950,000 m^3^/day, and serves 519,300, 2,420,000, and 3,452,600 inhabitants, respectively. The sampling campaign was conducted from 21 December to 25 December 2020. Auto-samplers, FC-9624 (GRASP Science & Technology Co., Ltd., Beijing, China) was used to collect time-proportional composite samples. Each auto-sampler was programmed to draw 500 mL influent per hour, and a composite sample was obtained by mixing the samples collected over a 24-h period. All samples were collected in 1 L amber glass bottles and immediately carried to the laboratory in a frozen state and stored at −20 °C until analysis within two days.

### 2.3. Sample Preparation

The extraction method for different classes of antibiotics in wastewater samples was developed on the basis of previous method for determining sulfonamide metabolites or multiple classes of antibiotic parents in wastewater [32,34,35]. 

All water samples were filtered with a glass fiber filter (1 μm, Whatman, UK) to remove solid particles and then added with hydrochloric acid or sodium hydroxide to adjust pH values. In this study, the effect of pH on extraction efficiencies of target compounds in ultrapure water samples (load volume: 25 mL) was examined at two pH values (pH 3.0 and 7.0). 

After optimization of pH values and cartridges, 25 mL influent and 50 mL effluent were then extracted to evaluate extraction recoveries and matrix effects. The investigated SPE cartridges were preconditioned with 3 mL methanol, followed by 3 mL of acidified ultrapure water (pH 3.0). Subsequently, deuterated internal standards (5 ng) were added to filtered water samples for quantification. The spiked samples were loaded into the cartridges at a flow rate of 1 mL/min. Following passage of the samples, the cartridges were washed with 3 mL of water-methanol (90:10, *v*/*v*) to remove weakly bound impurities (for ultrapure and effluent water samples, this step was omitted). After drying under vacuum for about 20~30 min, the cartridges were then eluted with 2 mL of methanol containing 1% ammonia into a 10 mL glass tube. The eluates were dried under a gentle flow of nitrogen to a volume of ~50 µL and then reconstituted to 500 µL with water-methanol (50:50, *v*/*v*) solution. A further filtration step was performed by a 0.22 µm centrifugal filter (Cellulose Acetate, Costar, Washington, DC, USA). The final aliquots were transferred into 2 mL amber vials (CNW Technologies GmbH, Düsseldorf, Germany) and stored at 4 °C until UPLC-MS/MS analyses.

### 2.4. Liquid Chromatography and Mass Spectrometry

Analysis was carried out using a Waters ACQUITY UPLCTM system (Waters, Milford, MA, USA). Separation of target antibiotics and their metabolites was achieved with a Waters ACQUITY UPLC BEH C_18_ column (1.7 µm; 2.1 mm × 100 mm). The injection volume was 2 µL (full loop). The column was maintained at 40 °C and the sample room temperature was 4 °C. 10 mM ammonium acetate in ultrapure water containing 0.1% formic acid (*v*/*v*) (A) and methanol containing 0.1% formic acid (*v*/*v*) (B) were used as mobile phases. The flow rate was 0.3 mL/min. The separation of antibiotics was achieved with a gradient program described as follows: the mobile phase ratio of A:B was 80:20 at 0 min, 10:90 at 3 min and maintained for 0.2 min, 80:20 at 3.3 min and maintained for 2.5 min for column equilibration.

Mass spectrometry was performed using a Waters Xevo TQ-XS (triple-quadrupole) detector equipped with an electrospray ionization source (Waters, Milford, MA, USA). The mass analyzer was operated in positive ionization mode and the optimized parameters were as follows: source temperature, 150 °C; desolvation temperature, 500 °C; capillary voltage, 1.0 kV; desolvation gas flow, 1000 L/h; cone gas flow, 150 L/h; and Nebuliser, 7.0 bar. Quantitative analysis was performed in multiple reaction monitoring mode. MS/MS parameters for the analytes, including their parent and product ions, cone voltage, and collision energy were summarized in Table S3.

### 2.5. Method Validation

Analytes were quantified using deuterated compounds as IS. When an analyte specific IS was unavailable, surrogate internal standards were chosen based on their ability to correct for recovery losses following SPE. Calibration curves were established by injecting standard solutions prepared from the standard mixtures with the addition of an identical amount of IS. Calibration curves were constructed by calculating the ratios between the peak area of target antibiotics and the corresponding IS peak area using linear regression analysis (y = ax + b). For each analyte, an eight-point calibration curve in the range of 0.1~100 ng/mL was generated with a satisfactory correlation coefficient (r^2^ > 0.99).

Instrumental detection limit (IDL) and instrumental quantification limit (IQL) for each analyte were determined as the minimum detectable amount of analyte, giving a signal to noise ratio of 3 and 10, respectively. The method detection limits (MDL) and method quantitation limits (MQL) were estimated by determining S/N of the lowest measured concentrations and extrapolating to S/N values of 3 and 10, respectively [40,41]. 

The matrix effect was determined according to Matuszewski et al. [42]. Four sets of samples were prepared as follows: Set A was prepared by extracting wastewater samples without spiking; Set B was prepared by extracting wastewater samples and then reconstituting the dried extracts with standard and IS; Set C was prepared by spiking the filtered wastewater before extraction with standard and IS; and Set D was prepared by mixture solution dissolved in water-methanol (50:50, *v*/*v*). The matrix effect was obtained by dividing the peak areas from Set D by the peak areas from the difference between Set B and Set A. The signal of the analyte is enhanced if matrix effect > 0, whereas the signal of analyte is suppressed if matrix effect < 0. The recovery was determined by diving the peak areas from the peak areas from Set D by the peak areas from the difference between Set C and Set A.

The instrument intra-day precision was assessed as the relative standard deviation (RSD) of ten repeated injections of a 10 µg/L standard (IS level:10 µg/L). The instrument inter-day precision was assessed as the RSD of 10 µg/L standards (IS level:10 µg/L) analyzed throughout five days. 

The precision of the method was assessed by determining the solid phase extraction recoveries. A series of extraction experiments were conducted by spiking the target analytes with IS into ultrapure water samples and processed in triplicate.

### 2.6. Stability Tests

To estimate the stability of parent antibiotics (SPY, SMX, RTM, ATM, TMP, CTM, LIN, labeled as Group 1) and their metabolites (N-SPY, N-SMX, N-RTM, Des-ATM, 4-TMP, labeled as Group 2) under different sample storage conditions, two stability batch tests were performed as the following. In the first batch (Batch 1), 50 mL aliquots of influent wastewater were spiked with 50 µg/L of Group 1 and Group 2 mixture solutions, respectively, and stored in polytetrafluoroethylene bottle for 48 h under 4 °C. Another batch (Batch 2), 100 mL aliquots of influent wastewaters were spiked with the same concentration of the two group mixture solutions, respectively. After spiking, wastewater samples were well homogenized by shaking and divided into 10 identical aliquots of 10 mL. They were then stored in polytetrafluoroethylene bottle under different frozen conditions: −20 °C and −80 °C. The stability tests were performed in the dark. The pH value in the two batches were adjusted to 3. Aliquots of 1 mL were taken at the beginning of every batch test (time 0) and after 1, 2, 4, 8, 12, 24, and 48 h in Batch 1 and after 1, 2, 4, 7, 14 and 30 day for Batch 2. After thawing (as needed), the aliquots were filtered through 0.22 µm cellulose acetate filters and injected (2 µL) into the LC-MS system. Internal standards were spiked to the filtered samples just prior to the instrument detection to reduce instrument interference. Each sampling procedure was performed three times.

Time 0 samples were analyzed immediately after grouping and the concentration of time 0 samples was regarded as the initial value. The concentration of each time point was divided by the initial value to obtain a residual concentration percentage value in order to assess the degradation tendency of each analyte.

### 2.7. Blank Analysis

In addition, to check whether there was any potential contamination during the sample preparation stage and instrument analysis, ultrapure water blanks (pH = 3) were spiked only with IS (procedural blank) and used to check for any possible background concentration of target analytes. None of the target analytes was detected in these spiked blanks, suggesting that there was no contamination during the experiments.

## 3. Results and Discussion

### 3.1. UPLC-MS/MS and SPE Method Optimization

A positive ionization mode was chosen due to its high sensitivity to all target antibiotics and their metabolites. The precursor ion and the highest characteristic product ions were listed in Table S3. 

Efficient mobile phases and elution gradient were desired to separate target compounds with different polarities. Three different mobile phase combinations, such as 0.2% formic acid with 2 mM ammonium acetate in ultrapure water and acetonitrile (Conbination-1), ultrapure water containing 0.1% formic acid and 0.1% formic acid in methanol and acetonitrile (1:1, *v*/*v*) (Conbination-2) and 0.1% formic acid with 10 mM ammonium acetate in ultrapure water and 0.1% formic acid in methanol (Conbination-3), were investigated to optimized chromatographic performance. Poor peak shapes of RTM, N-RTM and CTM were observed in Conbination-1 (Figure S2). Conbination-2 produced a slight increase in signal intensity for TMP, 4-TMP and Des-ATM relative to Conbination-3. However, the signal intensities of ATM and RTM in Conbination-2 were about half in Conbination-3 (Figures S3 and S4). In addition, Conbination-3 yielded better chromatographic peak shapes for most compounds than Conbination-2. Finally, 10 mM ammonium acetate in ultrapure water containing 0.1% formic acid (*v*/*v*) (mobile phase A) and 0.1% formic acid in methanol (mobile phase B) were chosen as the mobile phases for routine UPLC-MS/MS analytes for the target compounds.

**Figure 1:**
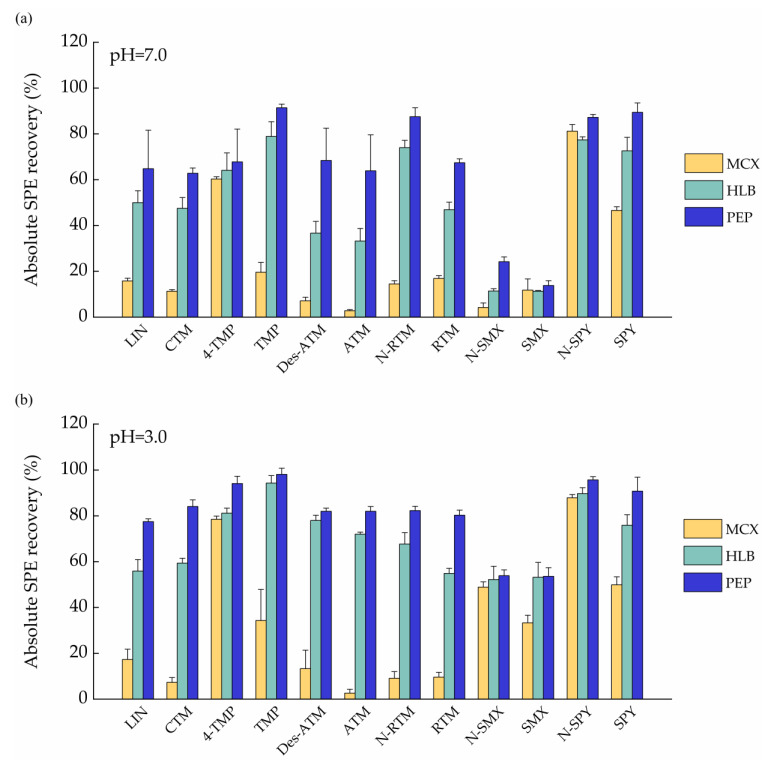
Absolute SPE recoveries of target analytes in ultrapure water samples enriched by various SPE cartridges (MXC, HLB, PEP) under different pH values. (**a**) pH = 7.0; (**b**) pH = 3.0.

In this study, three different SPE cartridges, namely HLB (3 mL, 60 mg, Waters, Milford, MA, USA), MCX (3 mL, 60 mg, Waters, Milford, MA, USA) and PEP (3 mL, 60 mg, Agela Technologies, Tianjin, China) were investigated to achieve an acceptable recovery for all the target analytes. Experiments were conducted using 25 mL ultrapure water sample spiked at 5 ng of each analyte under acidic (pH 3.0) and neutral conditions (pH 7.0). Figure 1 showed that MCX cartridges yielded lower extraction efficiencies for all target analytes than those by HLB and PEP cartridges under both acidic and neutral conditions. These might be explained by cation exchange cartridge (MCX) display good absorption to basic substances. For HLB and PEP cartridges, the extraction efficiencies for all targets under pH = 3.0 were higher than those under pH = 7.0 except for N-RTM. The results are similar with those reported in the previous literature [32]. Under acidic condition (pH = 3), the extraction efficiencies for five analytes (SPY, RTM, N-RTM, CTM, LIN) of PEP cartridges were higher than those of HLB (Figure 1b). In addition, no significant differences were observed in extraction efficiency for other analytes between HLB and PEP cartridges. On the basis of the obtained results, in particular the good compromise in extraction efficiency, PEP cartridges with acidic condition were selected for further validation as they provided more consistent results compared to Oasis HLB and MCX.

### 3.2. Method Validation

#### 3.2.1. Linearity and Range

Calibration curves were established with a satisfactory correlation coefficient for all compounds (r^2^ > 0.99), as presented in Table 1. Calibration curves of all targets ranged from 0.1 ng/mL to 100 ng/mL (the concentration gradient was at 0.1, 0.5, 1, 5, 10, 20, 50, 100 ng/mL).

#### 3.2.2. Detection and Quantification Limits

The instrumental detection limit (IDL) and instrumental quantification limit (IQL) were estimated from the lowest point of the calibration curve by giving a signal to noise ratio of 3 and 10, respectively. The IDL and IQL values for the target compounds ranged from 0.002 ng/mL to 0.07 ng/mL and 0.008 ng/mL to 0.2 ng/mL, respectively (Table S3). Method detection (MDL) and quantitation limits (MQL) were defined as analyte responses giving a signal to noise ratio of 3 and 10, respectively, and were estimated from extracted samples. Based on extrapolating, we calculated the MDL for all the target compounds ranged from 0.1 ng/L to 1.5 ng/L for the influent, 0.03 ng/L to 0.7 ng/L for the effluent, respectively. The MQL ranged from 0.2 ng/L to 5.0 ng/L for the influent, 0.2 ng/L to 2.4 ng/L for effluent, respectively (Table 1). These MDL were sufficiently low for quantification of all compounds in influent and effluent wastewater samples.

#### 3.2.3. Instrument and Method Precision

Instrument and method precision were calculated by the relative standard deviation (RSD) according to replicated determinations. The instrument intra-day and inter-day precision was analyzed by repeated injections of a 10 µg/L standard and the relative standard deviation (RSD) was less than 9.2% and 11.4%, respectively. Method intra-day and inter-day precision for all compounds was less than 4.9% and 8.2% RSD (Table 1).

#### 3.2.4. SPE Recovery

Table 2 shows absolute and relative SPE recoveries for the analytes in different water samples using the PEP cartridge under pH 3.0. The absolute recoveries for all the target analytes in the influents and effluents ranged from 73.3 to 95.4% and 72.0 to 102.7% except for SMX (57.3%) and CTM (58.5%). The relative SPE recoveries of all the target analytes were in the range between 83.0% and 104.4%, and the RSD was below 9.1%, indicating a good precision of the SPE procedure.

#### 3.2.5. Matrix Effect

As mentioned in Section 2.4, matrix effects were calculated by dividing the peak areas between Set B and Set A. In influents, all analytes were suppressed by the matrix (−0.7% to −28.7%), with SMX, N-SMX and TMP the most affected. Similar results were also found in effluents for most targets (−27.6% to −1.7%) except for SPY, RTM, N-RTM, Des-ATM and 4-TMP (1.4% to 4.8%) (Table 2).

### 3.3. Stability of Antibiotics and Metabolites

**Figure 2:**
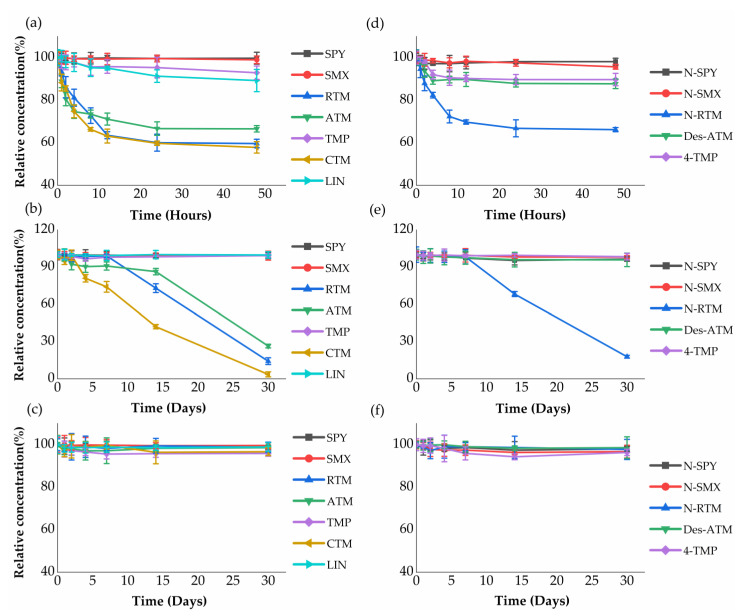
Analytes’ stability in wastewater kept under different conditions: (**a**–**c**) parents antibiotics stability profiles in wastewater kept at pH 3 and 4 °C for 48 h, pH 3 and −20 °C for 30 d, and pH 3 and −80 °C for 30 d; (**d**–**f**) metabolites stability profiles in wastewater kept at pH 3 and 4 °C for 48 h, pH 3 and −20 °C for 30 d, and pH 3 and −80 °C for 30 d.

Stability of antibiotics metabolites was performed at 4 °C (48 h), −20 °C (30 d) and −80 °C (30 d) to evaluate their potential degradation during sampling and storage period. The relative concentrations of sulfonamides parents (SPY and SMX) and corresponding metabolites (N-SPY and N-SMX) dropped by less than 5% after 48 h and 30 d, indicating these compounds were relatively stable in wastewater at 4 °C, −20 °C and −80 °C. This observation may be attributed to aromatic rings and double bond functional groups in sulfonamides, which are strong and highly resistant to biodegradation [43]. Similar results were also observed in other antibiotics (TMP and 4-TMP), with the relative concentration percentage above 90% (Figure 2a,b), since the structure of TMP and 4-TMP were similar with sulfonamides compounds.

Macrolides parents and corresponding metabolites were also stable after 30 day at −80 °C (Figure 2c,f). As shown in Figure 2a,b,d,e, macrolides metabolites, N-RTM and Des-ATM were relatively more stable than their corresponding parents (RTM and ATM). In contrast, the different degradation rates were observed in macrolides parents (RTM, CTM and ATM). Zero and first-order kinetics models were applied to evaluate the degradation of macrolides parent antibiotics (Table 3). Both zero and first-order kinetics exhibited low stability during the experiment period for RTM and CTM at 4 °C and −20 °C. The degradation rates of RTM were similar to CTM with a half-life of 69.3 h (95% CI: 40.8~231.0 h) at 4 °C and 10.8 d (95% CI: 7.8~17.8 d) at −20 °C following the first-order kinetic model, respectively. Compared to RTM and CTM, ATM showed a longer half-life of 115.5 h (95% CI: 57.0~∞ h) at 4 °C and 16.5 d (95% CI: 11.4~30.1 d) at −20 °C. These results may be explained by the fact that macrolides are adsorbed to wastewater biomass or glass tube walls due to hydrophobic interactions [44]. This is expected due to their high lipid-water partition coefficient [45].

Although noticeable degradation of macrolides parent antibiotics and their metabolites was observed at 4 °C and −20 °C, significant decrease in their concentrations is not expected to occur during the sampling (<24 h). After sampling, the wastewater samples, if not stored at −80 °C, should be analyzed as soon as possible (maximum within a couple of days). For samples that cannot be analyzed in a short period of time after sampling, they must be kept at −80 °C. Further research is required to explore the effect of biofilms, as well as aerobic and anaerobic conditions since these parameters have potential impacts on the stability of analytes in sewers [46].

### 3.4. Application to Real Wastewater Samples

The optimized method was applied to influent wastewater collected from three Beijing wastewater treatment plants from 21 December to 25 December 2020. The detection frequency, range, median, arithmetic mean concentrations, standard deviations, and apparent removal rate are presented in Table 4.

In influent samples, all selected antibiotics and their metabolites were detected in a concentration above MDL, demonstrating the sensitivity of this method. Among all analytes investigated in this study, macrolides were detected at the highest concentrations (6.0~1755.4 ng/L), with CTM being the dominant compound. Concentrations of CTM in the influent samples were in the range of 874.6~2908.4 ng/L, which is much higher than those reported in Zhang et al. [47] (1.2~661.4 ng/L). The concentrations of Des-ATM and ATM in influent ranged from 317.2 ng/L to 639.8 ng/L and 969.2 ng/L to 1755.4 ng/L, respectively. The ratio of Des-ATM to ATM ranged from 0.27 to 0.39 with a median of 0.36, which is at a similar level with the ratio of 0.27 (after oral administration) in a previous ileostomy fluid study [48]. All four sulfonamides (SPY, N-SPY, SMX and N-SMX) were detected, ranging from 87.2 ng/L to 772.2 ng/L in the influent. The mean concentration of N-SPY and N-SMX were detected up to 326.0 ± 56.3 ng/L and 498.7 ± 151.4 ng/L, higher than their parent compounds, SPY (110.9 ± 19.7 ng/L) and SMX (139.9 ± 29.1 ng/L). Metabolites concentrations higher than those of parent antibiotics were also found in two Switzerland municipal wastewater treatment plants [44]. This may be explained by retransformation between sulfonamides parents and their metabolites [44].

Macrolides metabolites (N-RTM and Des-ATM) were detected in most effluent samples with the same detection frequency (91.7%). The detection frequencies of N-SPY and N-SMX were 66.7%, while 4-TMP concentrations were below MDL in all effluent samples. Among the five analyzed metabolite compounds in effluents, Des-ATM (85.2 ± 92.5 ng/L) had the highest mean concentration, followed by N-SPY (84.6 ± 64.3 ng/L) and N-RTM (70.3 ± 73.0 ng/L). Removal rates of most selected parent compounds were high with mean apparent removal rates above 90% except for SMX and TMP. The apparent removal rate of SPY and SMX were 92.8 ± 9.5% and 87.6 ± 19.7%, which are higher than those reported in Gao et al. [49] (SPY: 39%, SMX: 40%). Besides, negative removal rates of RTM were reported previously [49] but were not observed in this study. The apparent removal rates of most metabolites (N-SMX, N-RTM and Des-ATM) were similar to their corresponding parents, except for 4-TMP and N-SPY (Table 4). 4-TMP was completely removed by wastewater treatment processes, whereas the removal of N-SPY (mean: 72.6 ± 21.4%) was significantly lower than those of N-SMX, N-RTM and 4-TMP (*p* < 0.05). To our knowledge, this is the first report on N-SPY, N-RTM, Des-ATM, and 4-TMP removal during wastewater treatment. As for N-SMX, only a few previous studies included it. The apparent removal rate of N-SMX (89.1 ± 8.7%) in this study is lower than that reported by Göbel et al. [44] (almost complete removal after secondary treatment).

## 4. Conclusions

A reliable and sensitive method was developed for the simultaneous determination of antibiotics and their human metabolites of different classes using the PEP cartridge coupled with ultra-high-performance liquid chromatography tandem mass spectrometry to further help evaluate health risk. The method detection limit were predominantly under 0.5 ng/L and 0.3 ng/L in influent and effluent wastewater, respectively. The overall analytical method was fully validated, obtaining satisfactory recovery (73.3~95.4% in influent and 72.0~102.7% in effluent for most compounds), accuracy and precision. Stability of antibiotics and their main human metabolites under different conditions was evaluated for guiding sampling and storage period. In summary, all analyzed metabolites and most parent compounds can be sampled at 4 °C and stored at −20 °C in a short time (7 d). If samples are stored for a long time, they should be kept at −80 °C. The method was successfully applied to wastewater samples from three Beijing WWTPs with all analytes detected in influent samples, demonstrating the sensitivity of this method. Macrolides was the most prevalent in influent, with CTM being the dominant compound. Removal rates of most analytes were high with mean apparent removal rates above 80% except for N-SPY. In further research, we will analyze more wastewater samples all over the city in China by this method to estimate the consumption of antibiotics and assess its environment risks to inform proper pharmaceutical control policy.

## Figures and Tables

**Table 1:** Inter/intra-day precision (RSD%), method precision (RSD%), linearity of calibration curves (R^2^), method detection limit (MDL), method quantitation limit (MQL).

Target Analytes	Curve (R^2^)	Range (ng/mL)	Instrument Precision		Method Precision		MDL (ng/L)		MQL (ng/L)	
			Intraday (*n* = 5)	Interday (*n* = 5)	Intraday (*n* = 5)	Interday (*n* = 5)	In ^a^	Ef ^b^	In ^a^	Ef ^b^
SPY	0.999	0.1–100	5.2	8.5	2.3	2.8	0.1	0.05	0.3	0.2
N-SPY	0.994	0.1–100	3.7	7.8	4.9	3.3	0.4	0.2	1.3	0.6
SMX	0.998	0.1–100	4.7	8.2	4.8	3.1	0.2	0.1	0.6	0.4
N-SMX	0.999	0.1–100	3.4	9.6	4.8	1.6	0.5	0.3	1.7	0.9
RTM	0.999	0.1–100	2.1	5.5	1.9	8.0	0.2	0.1	0.7	0.3
N-RTM	0.999	0.1–100	3.6	8.0	2.1	7.0	0.1	0.03	0.2	0.1
ATM	0.999	0.1–100	4.0	8.1	2.3	6.9	0.1	0.05	0.3	0.2
Des-ATM	0.999	0.1–100	6.7	10.1	3.8	5.9	0.1	0.04	0.2	0.1
TMP	0.999	0.1–100	3.7	4.1	0.8	8.2	0.1	0.03	0.3	0.1
4-TMP	0.999	0.1–100	2.6	2.3	0.5	7.6	0.2	0.1	0.7	0.2
CTM	0.999	0.1–100	2.8	11.4	2.4	8.1	1.5	0.7	5.0	2.4
LIN	0.999	0.1–100	9.2	10.8	1.3	5.6	0.5	0.2	1.6	0.8

^a^ In, Influent; ^b^ Ef, Effluent.

**Table 2:** Absolute and relative SPE recoveries of analytes in various environment samples using the PEP cartridge under extraction pH of 3.0, and matrix effects on analytical method performance.

Target Analytes	Absolute Recovery ^a^ Mean ± RSD ^b^ (%)			Relative Recovery ^a^ Mean ± RSD ^b^ (%)			Matrix Effects ^a^ Mean ± RSD ^b^ (%)	
	U W ^c^ (*n* = 3)	Influent (*n* = 3)	Effluent (*n* = 3)	U W ^c^ (*n* = 3)	Influent (*n* = 3)	Effluent (*n* = 3)	Influent (*n* = 3)	Effluent (*n* = 3)
SPY	90.8 ± 6.7	93.4 ± 3.7	82.8 ± 18.1	98.0 ± 0.3	90.4 ± 3.7	95.7 ± 2.8	−10.9 ± 5.3	2.7 ± 1.8
N-SPY	95.7 ± 1.5	92.7 ± 15.8	93.6 ± 4.6	96.1 ± 3.6	93.7 ± 3.5	91.7 ± 1.9	−7.6 ± 7.8	−7.8 ± 3.1
SMX	53.6 ± 7.1	77.6 ± 5.2	57.3 ± 12.2	97.2 ± 1.5	91.1 ± 3.7	92.9 ± 1.0	−28.7 ± 7.0	−27.6 ± 11.1
N-SMX	53.9 ± 4.6	73.3 ± 6.6	72.0 ± 2.4	98.0 ± 0.4	89.1 ± 9.1	98.0 ± 8.0	−26.5 ± 10.4	−18.0 ± 1.4
RTM	80.3 ± 2.7	76.3 ± 2.3	76.2 ± 7.4	96.1 ± 0.5	102.3 ± 3.3	92.0 ± 7.1	−6.0 ± 6.3	2.6 ± 9.4
N-RTM	82.2 ± 2.3	74.5 ± 3.2	77.3 ± 10.2	98.6 ± 0.1	99.8 ± 3.5	93.3 ± 7.5	−2.0 ± 5.2	4.8 ± 7.1
ATM	82.0 ± 2.6	87.0 ± 11.2	88.5 ± 3.8	98.9 ± 0.2	90.3 ± 6.9	88.3 ± 1.6	−14.4 ± 1.7	−2.1 ± 2.1
Des-ATM	82.0 ± 1.7	92.4 ± 5.6	83.1 ± 14.8	98.9 ± 2.6	96.0 ± 9.5	83 ± 12.6	−13.3 ± 6.6	1.4 ± 1.1
TMP	98.1 ± 2.8	92.4 ± 3.4	90.3 ± 5.9	99.6 ± 0.5	104.4 ± 2.0	95.1 ± 5.5	−20.1 ± 4.1	−5.6 ± 1.0
4-TMP	94.1 ± 3.4	95.4 ± 6.0	102.7 ± 3.7	98.7 ± 0.6	94.0 ± 3.2	89.2 ± 2.7	−3.1 ± 1.5	2.2 ± 1.1
CTM	84.1 ± 3.4	58.5 ± 4.0	76.8 ± 3.7	98.8 ± 0.4	98.5 ± 7.4	104.2 ± 5.0	−0.7 ± 0.9	−6.5 ± 4.6
LIN	77.5 ± 1.5	77.6 ± 3.7	82.9 ± 4.1	92.8 ± 3.3	104.1 ± 1.7	100.0 ± 3.0	−14.8 ± 6.9	−1.7 ± 4.2

^a^ The spike levels were at: ATM, Des-ATM, 5, 10, 2.5 μg/L; SPY, SMX, 5, 5, 5 μg/L; the other analytes: 5, 5, 2.5 μg/L in ultrapure water, influent, effluent, respectively. ^b^ RSD, Relative Standard Deviation.^c^ UW, Ultrapure water.

**Table 3:** Zero and first order kinetics to model macrolide antibiotics in laboratory wastewater under 4 °C and −20 °C.

Compounds	Degree (°C)	Zero Order		First Order Kinetics	
		Slope (%/h)	R^2^	Half-Life (h/Day)	R^2^
RTM	4	−0.74 (−1.3 to −0.15)	0.61	69.3 (40.8–231.0)	0.65
	−20	−2.9 (−3.7 to −2.1)	0.95	10.8 (7.8–17.8)	0.90
ATM	4	no significance	-	115.5 (57.8–∞)	0.50
	−20	−2.3 (−3.1 to −1.4)	0.91	16.5 (11.4–30.1)	0.87
CTM	4	−0.70 (−1.3 to −0.07)	0.55	69.3 (34.7–346.6)	0.60
	−20	−3.3 (−3.9 to −2.7)	0.97	6.3 (5.0–8.5)	0.95

**Table 4:** Statistics of target analytes concentrations (ng/L) in Beijing WWTPs (*n* = 24).

Target Analytes	Influent				Effluent				Removal ^c^ (%)
	DF ^a^ (%)	Mean ± SD ^b^	Med ^d^	Range	DF ^a^ (%)	Mean ± SD ^b^	Med ^d^	Range	
SPY	100	110.9 ± 19.7	104.4	87.2~137.4	66.7	7.2 ± 8.8	4.6	<MDL~23.1	92.8 ± 9.5
N-SPY	100	326.0 ± 56.3	311.7	277.8~351.2	66.7	84.6 ± 64.3	106.7	<MDL~153.0	72.6 ± 21.4
SMX	100	139.9 ± 29.1	131.1	103.0~190.0	66.7	17.0 ± 24.9	6.5	<MDL~66.0	87.6 ± 19.7
N-SMX	100	498.7 ± 151.4	461.4	302.2~772.2	66.7	61.0 ± 58.9	54.0	<MDL~167.7	89.1 ± 8.7
RTM	100	201.9 ± 51.6	195.1	118.0~304.6	100	13.1 ± 15.1	7.9	0.4~49.5	93.6 ± 7.3
N-RTM	100	9.0 ± 3.1	8.3	6.0~15.8	91.7	70.3 ± 73.0	53.8	<MDL~227.9	94.5 ± 3.7
ATM	100	1258.8 ± 245.1	1214.1	969.2~1755.4	100	28.0 ± 25.7	23.2	<MQL~71.6	97.6 ± 2.2
Des-ATM	100	439.9 ± 105.6	408.2	317.2~639.8	91.7	85.2 ± 92.5	40.5	<MDL~235.6	81.7 ± 18.0
TMP	100	143.8 ± 48.2	145.7	82.2~240.4	66.7	20.5 ± 17.0	26.3	<MDL~43.8	86.3 ± 11.1
4-TMP	100	1.9 ± 0.5	1.8	1.2~2.2	0	<MDL	<MDL	<MDL	100
CTM	100	1619.5 ± 560.2	1653.4	874.6~2908.4	100	53.5 ± 66.2	18.6	0.5~177.3	96.3 ± 4.6
LIN	100	251.2 ± 177.6	207.9	75.2~642.8	50	6.1 ± 9.3	0.2	<MDL~26.0	95.7 ± 6.7

^a^ DF, Detection frequency; ^b^ SD, Standard Deviation; ^c^ Apparent removal rate; ^d^ Med, Median.

## Data Availability

The data presented in this study are available in insert article or Supplementary Materials.

## Supplementary Materials

The following are available online at https://www.mdpi.com/article/10.3390/ijerph182010640/s1. Table S1: The selected analytes in this study; Table S2: The chemical characteristics of SPE cartridges used in this study; Table S3: Optimized instrument and MRM conditions of target analytes; Figure S1: The structures of the selected analytes in this study; Figure S2: Total-ion chromatogram of the selected analytes prepared in methanol/ultrapure water (1:1) under mobile phase: 0.2% formic acid with 2 mM ammonium acetate in ultrapure water and acetonitrile (Conbination1). The concentration of the analytes was 50 μg/L; Figure S3: Total-ion chromatogram of the selected analytes prepared in methanol/ultrapure water (1:1) under mobile phase: ultrapure water containing 0.1% formic acid and 0.1% formic acid in methanol and acetonitrile (1:1, v/v) (Conbination 2). The concentration of the analytes was 50 μg/L; Figure S4: Total-ion chromatogram of the selected analytes prepared in methanol/ultrapure water (1:1) under mobile phase: 0.1% formic acid with 10 mM ammonium acetate in ultrapure water and 0.1% formic acid in methanol (Conbination 3). The concentration of the analytes was 50 μg/L.
