# Supplementary material for: Simultaneous Determination of Seven Antibiotics and Five of Their Metabolites in Municipal Wastewater and Evaluation of Their Stability under Laboratory Conditions
Source: Int J Environ Res Public Health. 2021 Oct 11;18(20):10640. doi: 10.3390/ijerph182010640 (PMC8535447; doi:10.3390/ijerph182010640)
Supplement: Supplementary file 1 [file ijerph-18-10640-s001.zip › ijerph-1369492-supplementary.pdf]

# Supplementary Information

# Simultaneous Determination of Seven Antibiotics and Five of Their Metabolites in Municipal Wastewater and Evaluation of Their Stability under Laboratory Conditions

**Sheng Han [1], Xinyue Li [2], Hongmei Huang [1], Ting Wang [1], Zhenglu Wang [3], Xiaofang Fu [1], Zilei Zhou [4], Peng Du [5] and Xiqing Li [1,*]**

[1] Laboratory of Earth Surface Processes, College of Urban and Environmental Sciences, Peking University, Beijing 100871, China; hans@pku.edu.cn (S.H.); 1701214469@pku.edu.cn (H.H.); wangting7@pku.edu.cn (T.W.); fxiaof@pku.edu.cn (X.F.)

[2] Development Research Center of the Ministry of Water Resources of P. R. China, Beijing 100036, China; lixinyue@waterinfo.com.cn

[3] College of Oceanography, Hohai University, Nanjing 210098, China; 20190039@hhu.edu.cn

[4] Hubei Provincial Academy of Eco-environmental Sciences, Wuhan 430070, China; 1601214925@pku.edu.cn

[5] Beijing Key Laboratory of Urban Hydrological Cycle and Sponge City Technology, College of Water Sciences, Beijing Normal University, Beijing 100875, China; dup@bnu.edu.cn

[*] Correspondence: xli@urban.pku.edu.cn

**Table S1.** The selected analytes in this study.

| Groups | Parent Antibiotics | Deuterated analogs | Main Human Metabolite | Deuterated analogs | Reference |
|---|---|---|---|---|---|
| Sulfonamides | Sulfapyridine | Sulfapyridine-$d_4$ | $N^4$-Acetyl Sulfapyridine | $N^4$-Acetyl Sulfapyridine-$d_4$ | [1] |
| | Sulfamethoxazole | Sulfamethoxazole-$d_4$ | $N^4$-Acetyl Sulfamethoxazole | $N^4$-Acetyl Sulfamethoxazole-$d_4$ | [1] |
| Macrolides | Roxithromycin | Roxithromycin-$d_7$ | N-Demethyl Roxithromycin | Roxithromycin-$d_7$ | [2] |
| | Azithromycin | Azithromycin-$d_3$ | Descladinose Azithromycin | Azithromycin-$d_3$ | [3] |
| | Clarithromycin | Clarithromycin-N-$d_3$ | — | — | — |
| Others | Trimethoprim | Trimethoprim-$d_3$ | 4-Hydroxy Trimethoprim | 4-Hydroxy Trimethoprim-$d_9$ | [1] |
| | Lincomycin | Roxithromycin-$d_7$ | — | — | — |

[1] Petrie, B., Barden, R., Kasprzyk-Hordern, B., A review on emerging contaminants in wastewaters and the environment: current knowledge, understudied areas and recommendations for future monitoring. Water Res. 2015, 72, 3-27.
[2] Li, X. Q., Zhong, D. F., Huang, H. H., Wu, S. D., Demethylation metabolism of roxithromycin in humans and rats. Acta Pharmacologica Sinica. 2001, 22, 469-474.
[3] Luke, D. R., Foulds, G., Disposition of oral azithromycin in humans. Clinical Pharmacology & Therapeutics. 1997, 61, 641.

**Table S2.** The chemical characteristics of SPE cartridges used in this study.

| SPE cartridge | Abbreviation | Sorbent material | Surface area ($m^2/g$) | Pore size (A°) | Particle size (μm) |
|---|---|---|---|---|---|
| Cleanert ® PEP (3 mL, 60 mg) | PEP | Polystyrene and divinylbenzene | 700 | 80 | 30 |
| Oasis® MCX (3 mL, 60 mg) | MCX | N-vinylpyrrolidone and divinylbenzene bonding with sulfonate group | 786 | 84 | 31.9 |
| Oasis® HLB (3 mL, 60 mg) | HLB | Hydrophilic N-vinylpyrrolidone and lipophilic divinylbenzene | 810 | 83 | 29.8 |

**Table S3.** Optimized instrument and MRM conditions of target analytes.

| Antibiotics | Dwell time (s) | Parent ion (m/z) | Cone voltage (V) | Product ion (m/z) | Collision energy (eV) | Retention time (min) | Internal standard | IDL (ng/mL) | IQL (ng/mL) |
|---|---|---|---|---|---|---|---|---|---|
| SPY | 0.004 | 250.1 | 35 | 92.0* | 25 | 1.58 | SPY-$d_4$ | 0.004 | 0.01 |
| | | | | 156.0 | 15 | | | | |
| N-SPY | 0.004 | 292.0 | 16 | 133.5 | 22 | 1.79 | N-SPY-$d_4$ | 0.02 | 0.06 |
| | | | | 197.7* | 14 | | | | |
| SMX | 0.004 | 254.1 | 30 | 92.0* | 25 | 2.03 | SMX-$d_4$ | 0.007 | 0.02 |
| | | | | 156.0 | 15 | | | | |
| N-SMX | 0.003 | 296.0 | 25 | 133.6* | 20 | 2.33 | N-SMX-$d_4$ | 0.02 | 0.06 |
| | | | | 198.0 | 20 | | | | |
| CTM | 0.016 | 748.5 | 30 | 158.0* | 30 | 3.43 | CTM-N-$d_3$ | 0.002 | 0.008 |
| | | | | 590.2 | 20 | | | | |
| RTM | 0.016 | 837.6 | 40 | 158.1* | 35 | 3.47 | RTM-$d_7$ | 0.004 | 0.01 |
| | | | | 679.5 | 20 | | | | |
| N-RTM | 0.016 | 823.5 | 14 | 143.7* | 38 | 3.46 | RTM-$d_7$ | 0.003 | 0.009 |
| | | | | 665.5 | 22 | | | | |
| ATM | 0.003 | 749.5 | 30 | 158.2 | 40 | 2.61 | ATM-$d_3$ | 0.01 | 0.03 |
| | | | | 591.5* | 30 | | | | |
| Des-ATM | 0.004 | 591.5 | 62 | 115.5 | 34 | 1.77 | ATM-$d_3$ | 0.07 | 0.2 |
| | | | | 157.7* | 30 | | | | |
| LIN | 0.005 | 407.2 | 40 | 126.1* | 25 | 1.83 | RTM-$d_7$ | 0.02 | 0.06 |
| | | | | 359.3 | 20 | | | | |
| TMP | 0.004 | 291.3 | 30 | 123.0* | 30 | 1.78 | TMP-$d_3$ | 0.009 | 0.03 |
| | | | | 230.0 | 30 | | | | |
| 4-H-TMP | 0.004 | 307.0 | 26 | 138.6* | 16 | 1.89 | 4-H-TMP-$d_9$ | 0.003 | 0.01 |
| | | | | 180.7 | 18 | | | | |
| SPY-$d_4$ | 0.004 | 253.9 | 30 | 112.0 | 22 | 1.58 | - | - | - |
| SMX-$d_4$ | 0.004 | 257.9 | 36 | 159.7 | 14 | 2.03 | - | - | - |
| N-SPY-$d_4$ | 0.004 | 296.0 | 8 | 137.6 | 22 | 1.79 | - | - | - |
| N-SMX-$d_4$ | 0.003 | 300.2 | 45 | 137.0 | 25 | 2.33 | - | - | - |
| CTM-N-$d_3$ | 0.005 | 751.6 | 6 | 160.7 | 30 | 3.43 | - | - | - |
| RTM-$d_7$ | 0.016 | 844.7 | 52 | 157.7 | 34 | 3.47 | - | - | - |
| ATM-$d_3$ | 0.003 | 752.7 | 46 | 594.6 | 28 | 2.61 | - | - | - |
| TMP-$d_3$ | 0.004 | 294.1 | 4 | 229.9 | 24 | 1.78 | - | - | - |
| 4-H-TMP-$d_9$ | 0.004 | 316.1 | 2 | 138.6 | 20 | 1.89 | - | - | - |

* Product ion was used for quantification

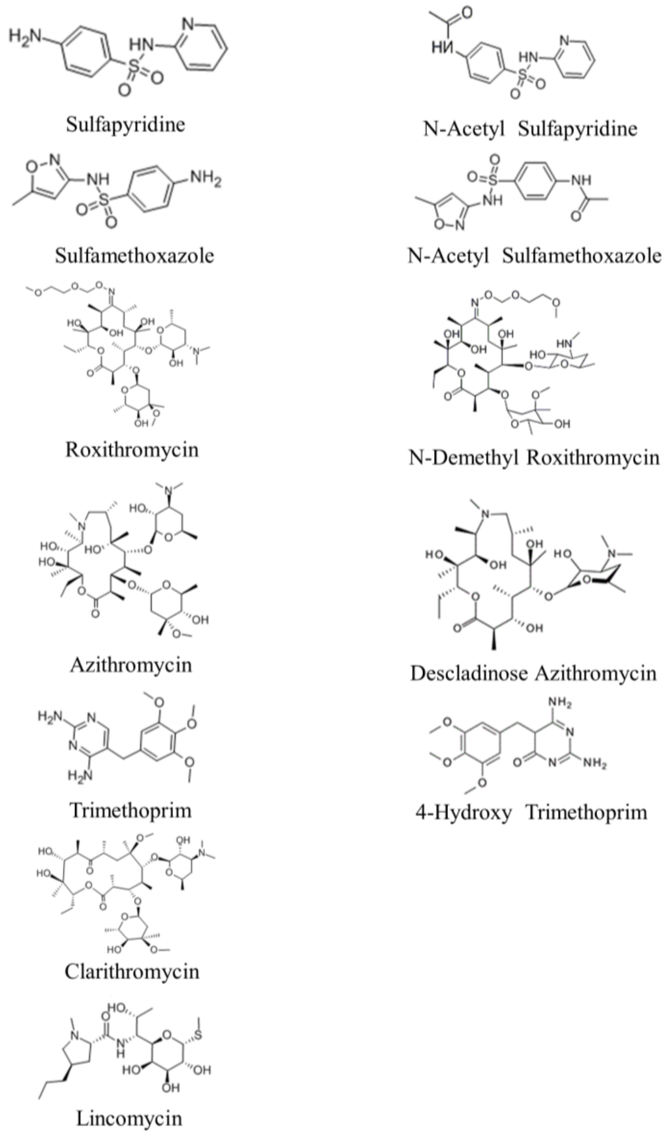


**Figure S1.** The structures of the selected analytes in this study.

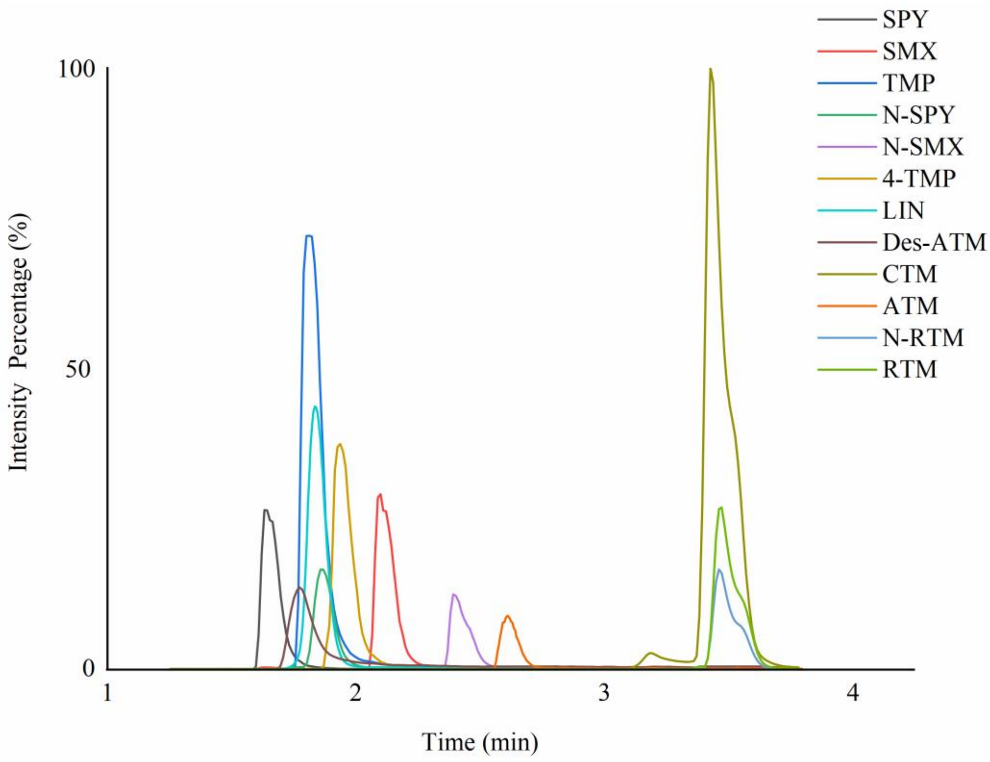


**Figure S2.** Total-ion chromatogram of the selected analytes prepared in methanol/ultrapure water (1:1) under mobile phase: 0.2% formic acid with 2 mM ammonium acetate in ultrapure water and acetonitrile (Conbination 1). The concentration of the analytes was 50 μg/L.

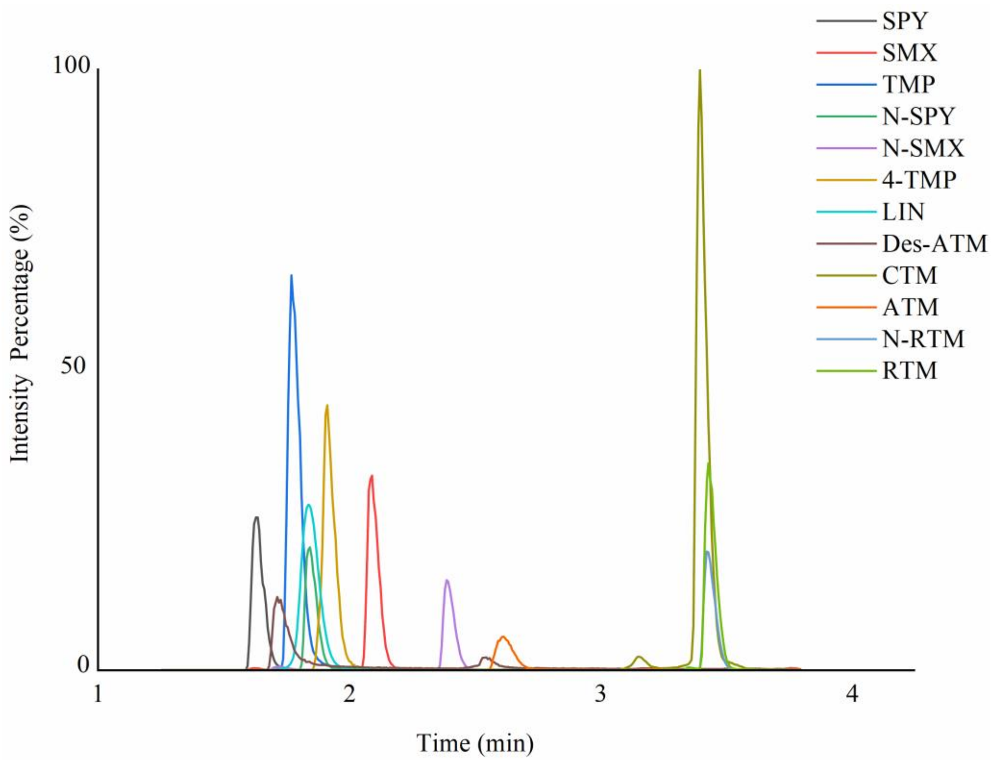


**Figure S3**. Total-ion chromatogram of the selected analytes prepared in methanol/ultrapure water (1:1) under mobile phase：ultrapure water containing 0.1% formic acid and 0.1% formic acid in methanol and acetonitrile (1:1,v/v) (Conbination 2). The concentration of the analytes was 50 μg/L.

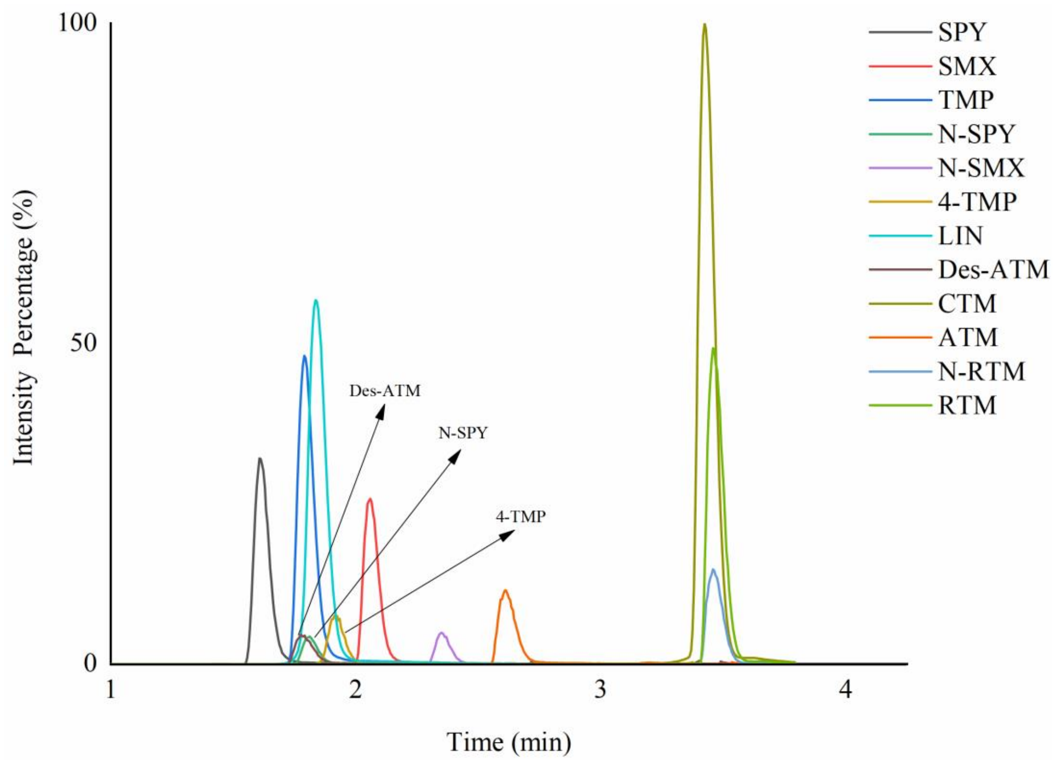


**Figure S4**. Total-ion chromatogram of the selected analytes prepared in methanol/ultrapure water (1:1) under mobile phase: 0.1% formic acid with 10 mM ammonium acetate in ultrapure water and 0.1% formic acid in methanol (Conbination 3). The concentration of the analytes was 50 μg/L.
